# Sphingosine kinase 1 is a potential therapeutic target for nasopharyngeal carcinoma

**DOI:** 10.18632/oncotarget.13014

**Published:** 2016-11-02

**Authors:** Wenhua Li, Jian Li, Yunchao Wang, Keqian Zhang, Ni Li, Zhiqiang Tian, Bing Ni, Huaizhi Wang, Zhihua Ruan

**Affiliations:** ^1^ Department of Oncology and Southwest Cancer Center, Southwest Hospital, Third Military Medical University, Chongqing 400038, PR China; ^2^ Institute of Hepatopancreatobiliary Surgery, Southwest Hospital, Third Military Medical University, Chongqing 400038, PR China Institute of Hepatopancreatobiliary Surgery, Southwest Hospital, Third Military Medical University, Chongqing 400038, PR China; ^3^ Institute of Immunology, PLA, Third Military Medical University, Chongqing 400038, PR China; ^4^ Department of Pathophysiology and High Altitude Pathology, Third Military Medical University, Chongqing 400038, PR China

**Keywords:** nasopharyngeal carcinoma, sphingosine kinase 1, therapeutic target, FTY720

## Abstract

Sphingosine kinase 1 (SPHK1) has been shown to be involved in the progression of various types of human cancers. We previously demonstrated that SPHK1 is overexpressed and associated with clinical stage, locoregional recurrence, distant metastasis and poor prognosis in nasopharyngeal carcinoma (NPC). However, the biological roles involving SPHK1 and its potential usefulness as a therapeutic target in NPC remain unknown. In this study, Blocking SPHK1 using siRNA or FTY720 (a SPHK1 inhibitor) significantly reduced proliferation and migration and increased cell cycle arrest and apoptosis in NPC cells. FTY720 also decreased SPHK1 activity, and overexpressing SPHK1 abrogated the FTY720-induced effects on cell viability. In addition, FTY720 sensitized NPC cells to radiotherapy by inhibiting SPHK1 activity *in vitro* and *in vivo*. Furthermore, high SPHK1 expression was associated with increased Ki-67 and p-Akt and decreased caspase-3 expression in human NPC specimens. These data suggest that SPHK1 might be a potential therapeutic target for NPC.

## INTRODUCTION

Nasopharyngeal carcinoma (NPC) is a head and neck epithelial malignant tumor that occurs frequently in Southeast Asia, especially in Southern China [1, 2]. Because of its highly invasive and metastatic features, NPC attracts more attention than other head and neck malignancies. Local recurrences and distant metastasis occur often, in more than one-third of NPC patients who reach the advanced stage of the disease [3]. The primary treatment of nasopharyngeal carcinoma is radiotherapy, but radioresistance remains a serious barrier that prevents successful treatment in a large number of patients [4]. There is therefore an urgent need to identify novel predictive markers of responsiveness to radiotherapy and to develop new therapeutic targets for NPC patients.

Sphingolipids are membrane lipids that are ubiquitously expressed in eukaryotic cells [5]. Bioactive sphingolipids, such as ceramide (Cer), sphingosine (Sph), and sphingosine 1-phophate (S1P), act as bioeffector molecules. These factors are involved in the regulation of various aspects of cancer pathogenesis, and they affect how therapies work by influencing cell proliferation, apoptosis, migration, senescence or responses to stressful conditions [6, 7]. Ceramide is considered to be an anti-cancer compound because it mediates and triggers cell growth arrest or apoptosis [8]. In contrast to ceramide, S1P plays a pro-survival function. S1P functions as a second messenger to regulate multiple cellular processes, and it can increase cell proliferation, resistance to apoptosis and angiogenesis via cell-surface G-protein-coupled receptors (S1PR1-S1PR5) [9]. The balance between pro-apoptotic ceramide and pro-survival S1P has been viewed as a sphingolipid rheostat that determines cell fate [10]. This balance is tightly regulated, mainly by sphingosine kinase 1 (SPHK1), which catalyzes the phosphorylation of sphingosine to produce pro-survival S1P [11].

An accumulating amount of data suggests that SPHK1 is associated with processes that are involved in cancer progression, such as cell oncogenesis, survival, metastasis and the neovascularization of the tumor microenvironment [12, 13]. It is well documented that the expression of SPHK1 is higher in various types of human cancers, including gastric cancer, glioma, head and neck squamous cell carcinoma, prostate cancer, breast cancer, and non-Hodgkin lymphomas, and that its expression is associated with the development and progression of the disease [14]. These data suggest that SPHK1 is a potential anti-cancer target [15]. For example, inhibiting SPHK1 using siRNA or pharmacological inhibitors significantly decreased proliferation, migration and angiogenesis in epithelial ovarian carcinoma [16], the SPHK1 inhibitor SK1-I regulated the ceramide-sphinogosine-S1P balance, suppressed proliferation and induced apoptosis in cholangiocarcinoma [17], and SPHK1 has been shown to be overexpressed and overactivated and to contribute to cetuximab resistance in human colorectal cancer models [18].

In our previous study, we found that SPHK1 protein levels are elevated in NPC and that high expression levels of SPHK1 are associated with clinical stage, locoregional recurrence, distant metastasis and poor prognosis in NPC [19]. Nevertheless, there are currently no data on the biological functions or potential roles of SPHK1 in NPC. In addition, the therapeutic effects of SPHK1 inhibitors in NPC remain unexplained. Therefore, the purpose of the present study was to investigate the *in vitro* and *in vivo* effects of targeting SPHK1 with siRNA or the pharmacological inhibitor FTY720 in NPC.

## RESULTS

### SPHK1 silencing inhibits NPC cell proliferation and induces cell cycle arrest and apoptosis

**Figure 1:**
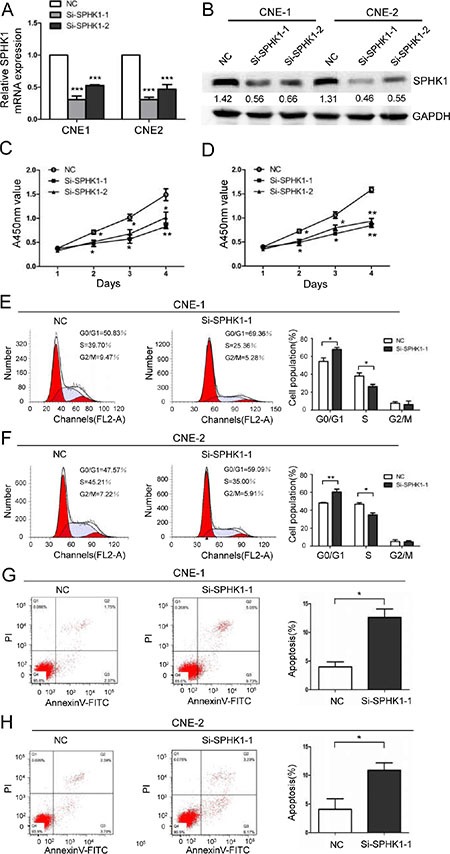
The downregulation of SPHK1 inhibits NPC cell proliferation and induces cell cycle arrest and apoptosis (**A**) CNE-1 and CNE-2 cells were transiently transfected with SPHK1 siRNAs using Lipofectamine. SPHK1 mRNA expression was analyzed using qRT-PCR at 24 h after transfection. β-actin was used as an internal control. (**B**) Western blot analysis showing the protein level of SPHK1 in CNE-1 and CNE-2 cells that were transfected with si-SPHK1-1 or si-SPHK1-2. After 48 h, SPHK1 levels were lower in the treated cells than in the negative control (NC). GAPDH was used as a loading control. (**C**, **D**) The proliferation of CNE-1 and CNE-2 cells was measured at the indicated times after transfection using a Cell Counting Kit-8 (CCK-8) kit. The results are presented as the means ± S.D. of values that were obtained in three independent experiments. Significance was calculated using Student's *t*-tests. (**E**, **F**) Flow-cytometric analysis of CNE-1 and CNE-2 cells that were infected with NC and SPHK1 siRNAs. (**G**, **H**) CNE-1 and CNE-2 cells were transfected with NC or SPHK1 siRNAs, stained with propidium iodide and Annexin V-fluorescein isothiocyanate (FITC), and analyzed using flow cytometry. Silencing SPHK1 significantly increased the rate of apoptosis in both cell types. *P < 0.05, **P < 0.01.

To determine the biological role of SPHK1 in the development and progression of NPC, we used two specific small interfering RNAs (siRNAs) against the SPHK1 mRNA, referred to here as si-SPHK1-1 and si-SPHK1-2. They significantly reduced the expression of the SPHK1 mRNA (Figure 1A) and protein (Figure 1B and Supplementary Figure S1) and significantly inhibited proliferation in both the CNE-1 and CNE-2 NPC cell line (Figure 1C and 1D). In addition, flow cytometry analysis revealed that the percentage of cells in the G0/G1 peak was clearly higher and the percentage of cells in the S peak was lower in the SPHK1 knockdown cells than in the negative control (NC) cells (Figure 1E and 1F). These results indicated that the silencing SPHK1 affects proliferation, potentially by regulating the G1-S phase transition. Moreover, the rate of apoptosis was significantly higher in the CNE-1 and CNE-2 cells that were treated with si-SPHK1 (Figure 1G and 1H).

### Sphingosine analogue FTY720 suppresses cell viability and induces cell cycle arrest and apoptosis in NPC cells by inhibiting SPHK1

**Figure 2:**
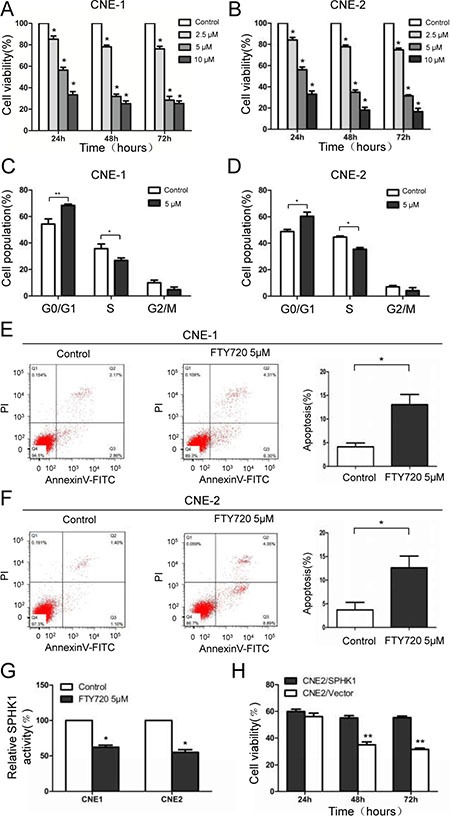
FTY720 induces the loss of cell viability, cell cycle arrest and apoptosis in NPC cells by inhibiting SPHK1 (**A**, **B**) Cell viability was measured using CCK-8 kits in CNE-1 and CNE-2 cells that were treated with varying doses of FTY720 for the indicated times. (**C**, **D**) Flow-cytometric analysis of the indicated NPC cells that were treated with FTY720. (**E**, **F**) CNE-1 and CNE-2 cells were treated with or without FTY720, stained with propidium iodide and Annexin V-fluorescein isothiocyanate (FITC), and then analyzed using flow cytometry. Apoptosis was noticeably accelerated following treatment with FTY720. (**G**) SPHK1 enzymatic activity was measured using a Sphingosine Kinase Activity Assay Kit. FTY720 significantly decreased SPHK1 activity in CNE-1 and CNE-2 cells. (**H**) CNE-2 cells that stably overexpressed SPHK1 (CNE-2/SPHK1) or an empty vector (CNE-2/Vector) were treated with 5 μM FTY720 for the indicated times, and cell viability was measured using CCK-8 kits. *P < 0.05, **P < 0.01.

To further assess the role of SPHK1 in NPC cells, we used FTY720 (also known as fingolimod), which can be used as a SPHK1 inhibitor [20], to block the endogenous activity of SPHK1 *in vitro*. FTY720 induced a dose-dependent inhibitory effect on cell viability in CNE-1 cells (Figure 2A), and a 5 μM dose achieved IC50 at approximately 30 hours after exposure. A similar decrease in cell viability was observed in CNE-2 cells (Figure 2B). In addition, FTY720 also induced G1-S arrest (Figure 2C and 2D) and apoptosis (Figure 2E and 2F) in CNE-1 and CNE-2 cells. Moreover, Sphingosine Kinase Activity Assay Kit results showed that FTY720 induced the rapid inhibition of SPHK1 enzymatic activity in CNE-1 and CNE-2 cells (Figure 2G). We previously constructed a lentiviral expression vector that stably overexpresses SPHK1 in pancreatic cancer cells [21]. To further confirm that the functional impact of FTY720 in NPC cells is mediated by the suppression of SPHK1, we reintroduced SPHK1 expression into FTY720-treated cells to analyze the antagonistic effect of FTY720. Interestingly, we found that overexpressing SPHK1 in CNE-2 cells rendered them less sensitive to FTY720 (Figure 2H).

### FTY720 significantly inhibits migration in NPC cells

**Figure 3:**
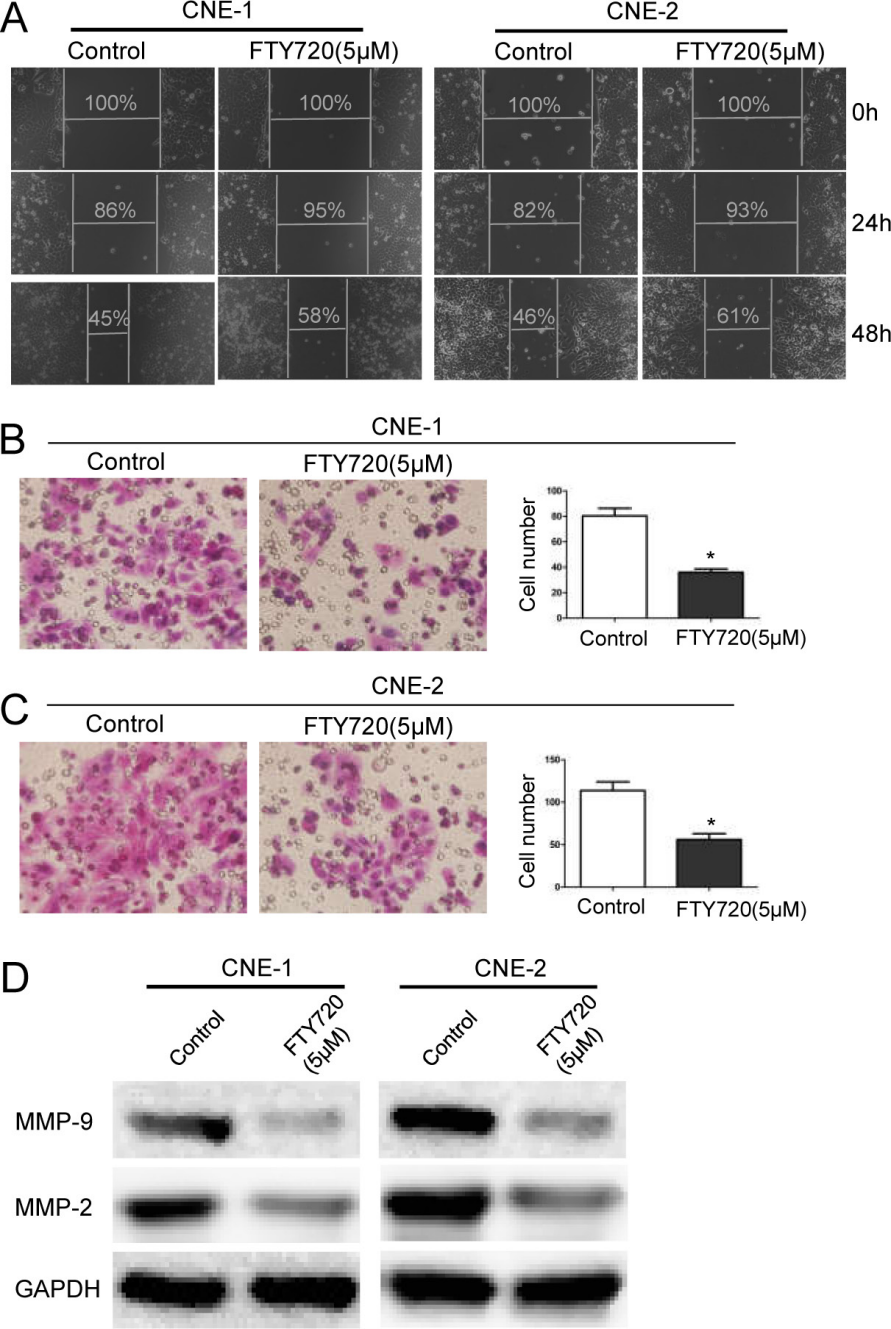
FTY720 inhibits migration in NPC cells (**A**) FTY720 suppresses cell migration in CNE-1 and CNE-2 cells. Images of wound healing assays were captured after 0, 24 and 48 h. (**B**, **C**) The inhibitory effect of FTY720 on cell migration was confirmed using transwell migration assays in CNE-1 and CNE-2 cells. The data are shown as the mean ± S.D. of three independent experiments. *P < 0.05. (**D**) The protein levels of MMP-2 and MMP-9 were evaluated using Western blot analysis in CNE-1 and CNE-2 cells that were treated with 5 μM FTY720 for 24 h. GAPDH was used as a loading control. FTY720 decreased the expression of MMP-2 and MMP-9 in NPC cells.

To determine the role of FTY720 in NPC cell migration, we first performed a wound healing assay. Treatment with FTY720 significantly decreased the migratory ability of CNE-1 and CNE-2 cells in a time-dependent manner (Figure 3A). In addition, consistent with the results of the wound healing assay, a transwell migration assay showed similar results, with a smaller number of cells penetrating the membrane in the FTY720-treated group than in the control group (Figure 3B and 3C).

Matrix metalloproteinases (MMPs) are gelatinases that play crucial roles in extracellular matrix turnover and cancer cell migration [22]. We therefore examined the expression of a selection of MMPs, including MMP-2 and MMP-9, after cells were treated with FTY720. Results showed that FTY720 significantly decreased the expression of MMP-2 and MMP-9 in CNE-1 and CNE-2 cells (Figure 3D). Taken together, these results demonstrate that FTY720 inhibits migration in NPC cells partially by downregulating MMPs.

### Effects of FTY720 on radiosensitivity in NPC cells cell lines

**Figure 4:**
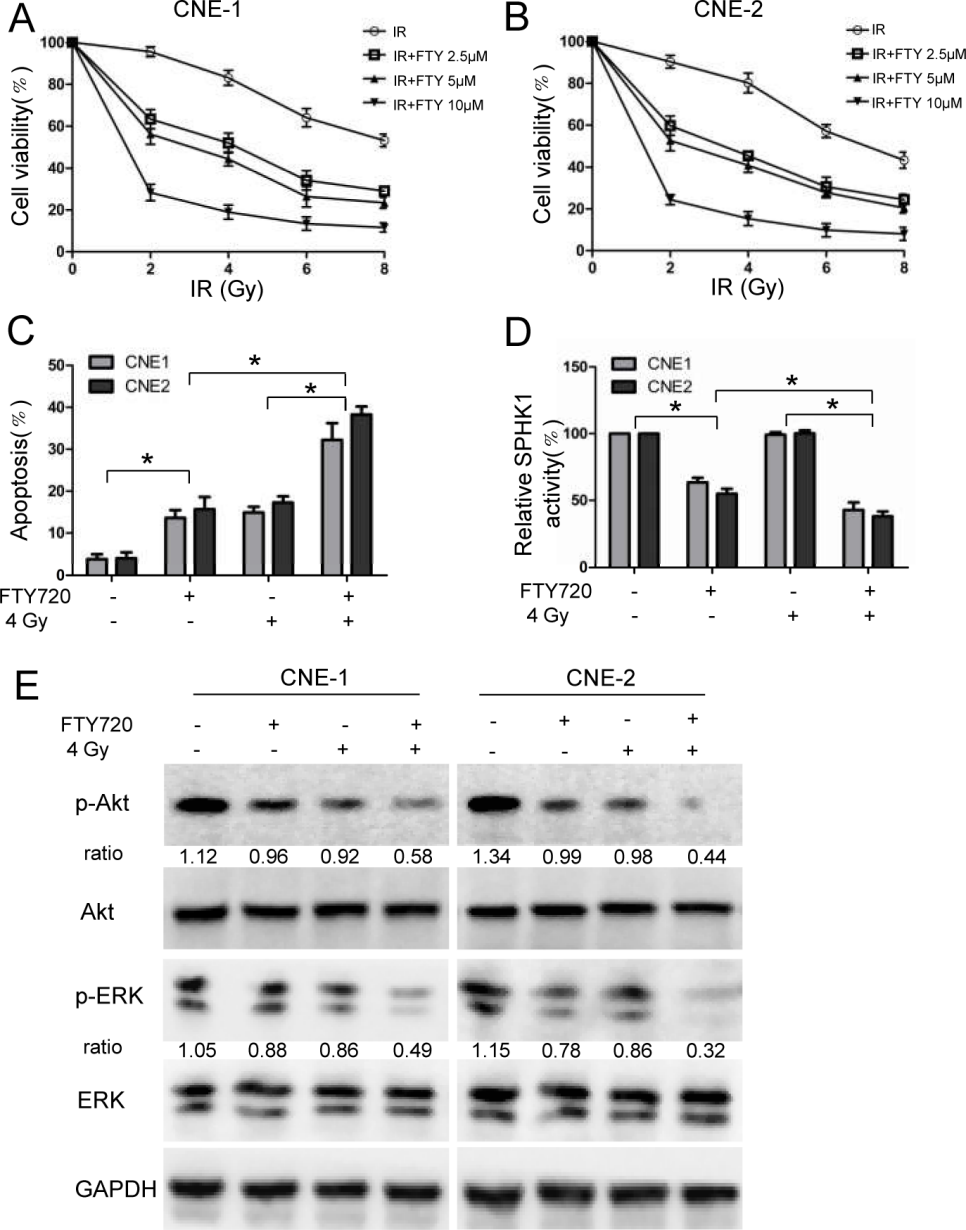
FTY720 sensitizes NPC cells to radiation (**A**, **B**) Cells were pretreated for 1 h with or without the indicated concentrations of FTY720 and exposed to different dose of radiation. The cells were then cultured for 48 h in the presence of FTY720. Cell viability was measured in CNE-1 (A) and CNE-2 (B) cells using CCK-8 kits. (**C**) The percentage of apoptotic cells in the cells that were treated with FTY720 (5 μM), radiation (4 Gy), or a combination of the two was measured using flow cytometric analysis. (**D**) FTY720 plus radiation significantly decreased SPHK1 enzymatic activity in CNE-1 and CNE-2 cells. SPHK1 activity was measured using a Sphingosine Kinase Activity Assay Kit. (**E**) Western blot analysis of protein expression levels in CNE-1 and CNE-2 cells that were treated with FTY720 (5 μM), radiation (4 Gy), or a combination of the two for 48 h. *P < 0.05.

We next assessed whether treatment with FTY720 could enhance radiation effects in NPC cells. Interestingly, we found that 2.5 μM, 5 μM, and 10 μM FTY720 successfully sensitized CNE-1 and CNE-2 cells to radiation (Figure 4A and 4B). These results suggest that FTY720 enhances the inhibitory effects of radiation on cell growth in NPC cells. To explore whether these growth-inhibitory effects are dependent on apoptosis, we used flow cytometry to determine the proportion of apoptotic cells after cells were treated with FTY720 and radiation. The results showed that in CNE-1 and CNE-2 cells, combining FTY720 with radiation induced substantially more apoptosis than using either treatment separately (Figure 4C), indicating a that these treatments exert a synergistic cell-killing effect. Figure 4D shows that adding FTY720 to NPC cells that were subjected to radiation significantly exacerbated the inhibition of SPHK1 activity, resulting in levels comparable to those in cells treated with FTY720 alone. However, treating NPC cells with radiation alone did not affect SPHK1 activity (Figure 4D).

To investigate the potential molecular mechanisms underlying the radio-sensitizing effects of FTY720, we evaluated the role of FTY720 combined with radiation on the activation of signaling pathways known to be involved in the regulation of cell survival and apoptosis. NPC cells were treated with radiation (4 Gy) and FTY720 (5 μM), and the expression levels of phospho-Akt and phospho-ERK1/2, a member of the MAPK family, were then determined using western blot analysis. The results showed that the combination treatment reduced the expression of phospho-Akt and phospho-ERK1/2 more than either FTY720 or radiation alone (Figure 4E).

### FTY720 sensitizes human NPC cells to radiation in nude mice xenografts

**Figure 5:**
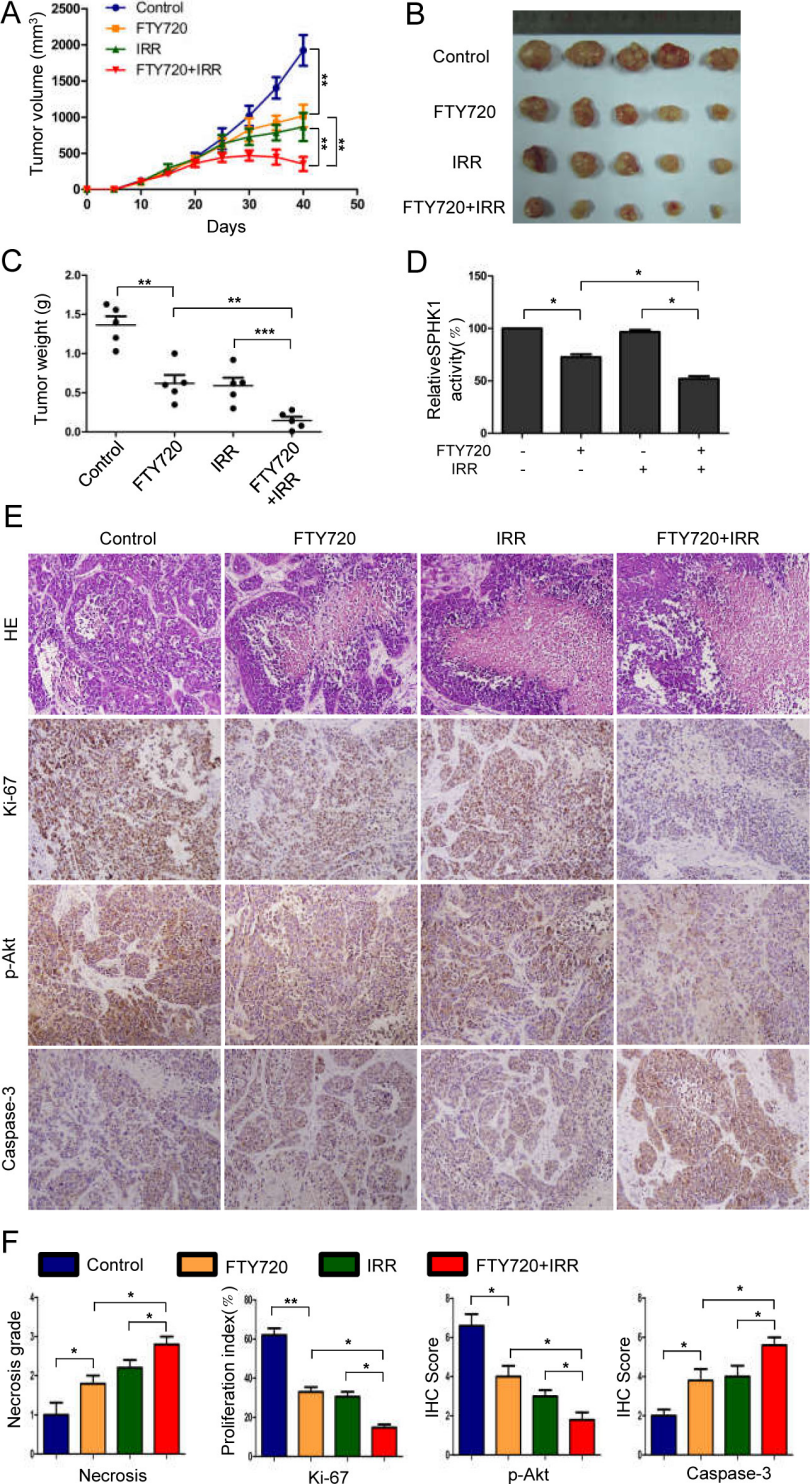
FTY720 sensitizes human NPC cells to radiation in nude mice xenografts (**A**) Twenty days after implantation, the mice were randomly divided into groups that received FTY720, radiation, or a combination of the two, as described in the Materials and Methods section. Tumor volumes were measured on the indicated days. The data points represented the mean tumor volumes ± SD. (**B**) A photograph of a representative xenograft is shown for each group. (**C**) Xenograft weights were measured at the time that the mice were sacrificed, and the mice in the combined treatment group showed the most significant reduction in tumor weight. (**D**) SPHK1 activity was measured in tissue extracts obtained from xenograft tumors. (**E**) Samples of xenograft tumors obtained from the four groups of mice were subjected to H&E and immunohistochemical staining for Ki-67, p-Akt and Caspase-3. (**F**) The necrosis grade and expression levels of the Ki-67, p-Akt and Caspase-3 proteins was calculated as necrosis grade and immunohistochemical staining scores. The error bars represent the standard error. *P < 0.05, **P < 0.01.

To determine whether FTY720 confers sensitivity to radiation to human NPC cells *in vivo*, we subcutaneously xenografted CNE-2 cells into nude mice. Twenty days after implantation, the mice were randomly assigned to different groups and treated for eighteen days with i.p. injections of 2.5 mg/kg/day FTY720 or/and 5 sessions of radiation (4 Gy) every 3 days. Two days after the last treatment, all of the mice were sacrificed. The growth curves of the xenografts showed that the combined treatment led to the most significant reduction in tumor volume (Figure 5A). Consistent with these results, FTY720 combined with radiation treatment resulted in a more dramatic reduction in the weight of the tumors than was observed in the other groups (Figure 5B and 5C). Sphingosine kinase enzymatic activity assays performed on tumor lysates revealed that while FTY720 significantly reduced SPHK1 activity in tumor cells, the combined treatment was much more effective (Figure 5D).

Based on data showing that loss of cell viability and proapoptotic effects were observed *in vitro* following treatment with a combination of FTY720 and radiation, we performed immunohistochemical staining for Ki-67, p-Akt and caspase-3 on tumor tissues that were collected from treated mice. In agreement with our *in vitro* findings, the combined treatment resulted in the most significant reduction in p-Akt expression and increase in caspase-3 expression (Figure 5E and 5F). In addition, the number of Ki-67-positive cells was also significantly lower in the combined treatment group than in the other three groups (Figure 5E and 5F). Moreover, the necrosis grade was low in the control, intermediate in the FTY720- or radiation-treated tumors and high in the combination-treated tumors (Figure 5E and 5F).

### High SPHK1 expression was associated with increased Ki-67 and p-Akt and decreased caspase-3 in human NPC specimens

**Figure 6:**
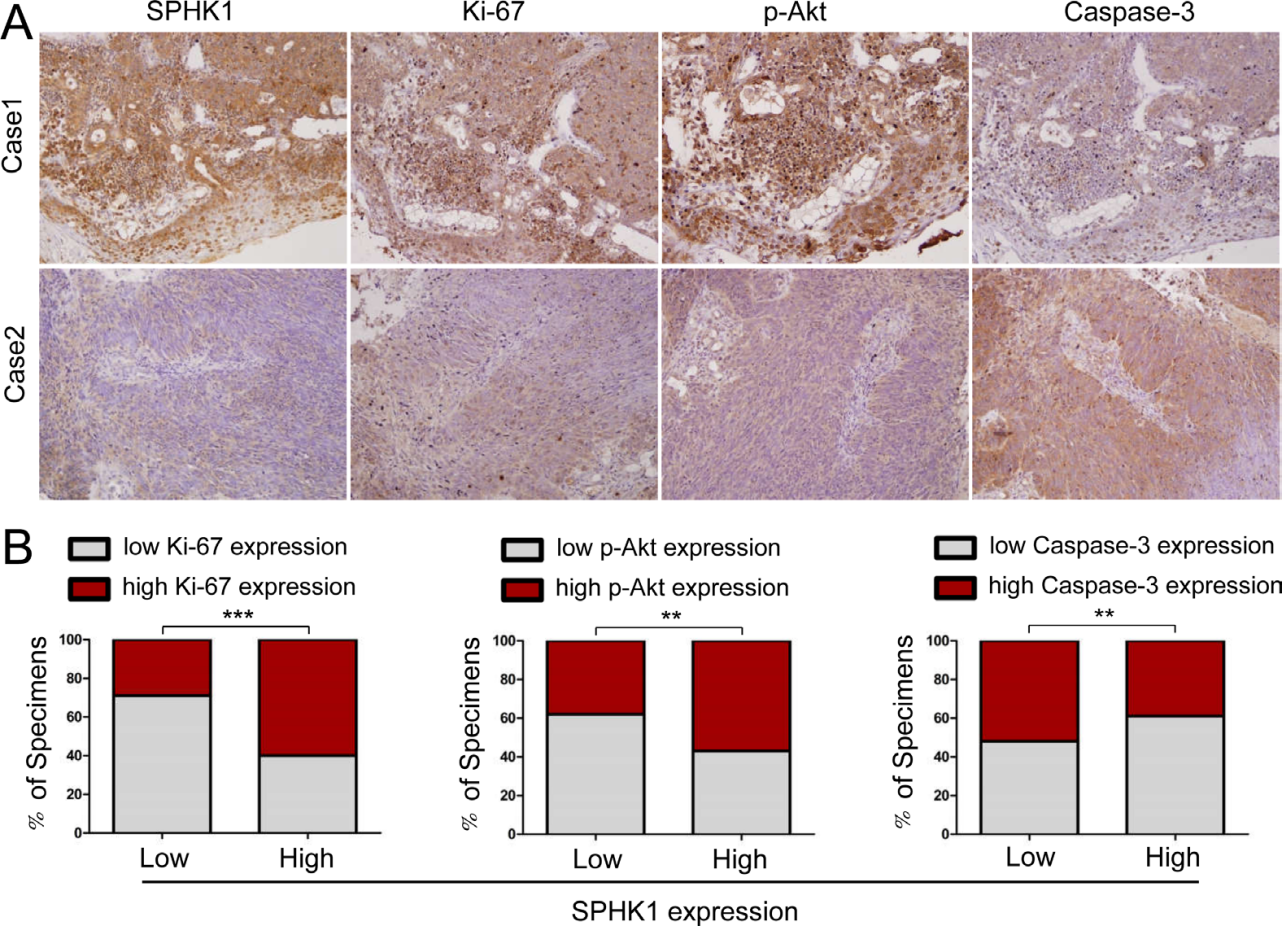
High SPHK1 expression was associated with increased Ki-67 and p-Akt and decreased caspase-3 expression in human NPC specimens (**A**) SPHK1 levels were associated with Ki-67, p-Akt and caspase-3 expression in 142 human NPC specimens. Two representative specimens that showed either a high or a low level of SPHK1 expression are shown. Original magnification, 200×. (**B**) The percentage of the specimens that showed a higher or lower level of SPHK1 expression than the level of Ki-67, p-Akt or caspase-3 are shown. **P < 0.01, ***P < 0.001.

In a previous study, we found that high levels of SPHK1 expression were associated with clinical stage, locoregional recurrence, distant metastasis and poor prognosis in 142 NPC patients [19]. Here, we show that SPHK1 is tightly associated with tumor growth and apoptosis. We hypothesize that there may be correlations between the expression of SPHK1 and the levels of survival and apoptosis related genes. To explore this hypothesis, we performed immunohistochemistry using samples from the same 142 NPC patients. We demonstrated that in these tumor tissues, the expression of SPHK1 was positively correlated with Ki-67 and p-Akt (Figure 6A and 6B). These results are consistent with our *in vitro* and animal model results. Moreover, high SPHK1 expression was inversely correlated with the expression of caspase-3, a member of the caspase family of enzymes (Figure 6A and 6B). Taken together, our *in vitro* and *in vivo* results demonstrate that high SPHK1 expression is associated with increased proliferation and reduced apoptosis in human NPC.

## DISCUSSION

Recent studies suggest that SPHK1 activation contributes to cancer progression by increasing oncogenic transformation, cell proliferation, resistance to therapies, and metastasis processes [15]. In this study, inhibiting SPHK1 using specific siRNAs or the pharmacological inhibitor FTY720 significantly decreased proliferation and induced cell cycle arrest and apoptosis in NPC cells. Moreover, we showed that FTY720 significantly decreased the intracellular enzymatic activity of SPHK1 and inhibited migration in NPC cells and the expression levels of MMP-2 and MMP-9. In addition, FTY720 sensitized NPC cells to radiotherapy by inhibiting SPHK1 activity *in vitro* and *in vivo*. When combined with radiation, FTY720 decreased the phosphorylation of Akt and ERK . Finally, high SPHK1 expression was associated with increased Ki-67 and p-Akt and decreased caspase-3 in human NPC specimens. These results provide new insights into the relationships between alterations in SPHK1 expression and activity and the development and progression of NPC.

SPHK1 is an oncogenic kinase that has been shown to be over-expressed in a diversity of human malignancies [14]. An accumulating amount of evidence demonstrates that SPHK1 is involved in cancer progression by contributing to oncogenic transformation, apoptosis, migration and resistance to treatment [12]. For example, SPHK1 is overexpressed in colon cancer, and the SPHK1/S1P pathway contributes to colon tumourigenesis [23]. Moreover, Song *et al.* have reported that SPHK1 is up-regulated in non-small cell lung cancer and involved in chemoresistance via PI3K/Akt/NF-κB signaling [24]. Our previous study showed that the expression of SPHK1 is elevated in NPC and correlated with a poor prognosis [19]. However, little is known about the biological function of SPHK1 in NPC. In the present study, we showed that downregulating SPHK1 using siRNAs affects a variety of cellular biological processes: it reduced cell proliferation and increased cell cycle arrest and apoptosis in NPC cells. To our knowledge, this study is the first to report that targeting SPHK1 has a direct effect on growth inhibition in NPC *in vitro* and *in vivo*.

FTY720 is a synthetic immunosuppressive compound that is derived as a metabolite from *Isaria sinclairii*. It has been extensively studied in renal transplantation [25]. Recently, FTY720 (Gilenya, Novartis) was FDA-approved as the first oral treatment for multiple sclerosis [26]. In cancer research, it has been investigated as an anticancer drug in various types of human cancers, including ovarian carcinoma [16], bladder cancer [27], and colorectal cancer [28]. So far, the feasibility of using this drug as a treatment for NPC has not been explored. In this study, we demonstrate that FTY720 decreased cell viability and migration and induced cell cycle arrest and apoptosis in NPC cells. An accumulating amount of evidence suggests that FTY720 could potentially be used as an anticancer drug, but the mechanisms underlying FTY720-induced cancer suppression have been reported to vary across different types of cancer [29, 30]. Specific targets that have been identified include SPHK1 [20, 31], protein phosphatase 2A [32], protein kinase C delta [33], and the phosphatidylinositol-3 kinase/Akt pathway [28]. In the present study, we showed that FTY720 acts as a SPHK1 inhibitor *in vitro* and *in vivo* and that the overexpression of SPHK1 in NPC cells was partially responsible for the suppressive role of FTY720. It is essential to further investigate alternative mechanisms of action that are modulated by FTY720 to develop additional novel treatment strategies for NPC.

In this study, we showed that the main cytotoxic effects of FTY720 were mediated by a decrease in cell survival and an increase in apoptosis. An additional cytotoxic effect of FTY720 was demonstrated in recent studies that showed that treatment with FTY720 potentiates autophagy. Autophagy plays an ambiguous role in cancer progression in that it can induce prolonged survival in cancer cells by conserving energy or it can lead to cell death [34]. FTY720-induced autophagy was reported to provide a protective effect against its other cytotoxic effects in experiments showing that inducing a blockade against autophagy enhanced FTY720-induced cell death [35, 36]. Moreover, autophagy-deficient murine embryonic fibroblasts were found to be more sensitive to FTY720-induced cytotoxicity [37]. However, another study showed that an FTY720-induced blockade of autophagy increased the pro-death activity of milatuzumab in mantle cell lymphoma [38]. Hence, previous studies have suggested that FTY720-associated effects on autophagy may be tissue type-dependent. Whether and how FTY720 is involved in autophagy in NPC has not been investigated and is currently being explored in our laboratory.

It has been suggested that SPHK1 plays a biologically significant role in chemo-/radio-resistance in different cancers [20, 39]. Elevated SPHK1 levels have been associated with resistance to imatinib in chronic myeloid leukemia (CML) and resistance to gemcitabine in pancreatic cancer cell. Conversely, inhibiting SPHK1 can sensitize CML and pancreatic cancer cells to the proapoptotic effects of imatinib and gemcitabine, respectively, apparently by reducing the S1P/ceramide ratio [40, 41]. *Sinha et al.* reported that inhibiting SPHK1 sensitized head and neck squamous cell carcinoma to radiation-induced cytotoxicity *in vitro* and *in vivo* [42]. In this study, we found that FTY720 effectively sensitized NPC cells to radiotherapy by inhibiting SPHK1 activity *in vitro* and *in vivo*. To our knowledge, this is the first study to link SPHK1 to radiosensitivity in NPC cells. Moreover, FTY720 significantly reduced the phosphorylation of Akt and ERK when in combined with radiation. ERK is frequently activated in NPC and is widely known to be involved in cellular growth, apoptosis and radioresistance [43, 44]. Finally, high SPHK1 expression was associated with increased Ki-67 and p-Akt and decreased caspase-3 expression in human NPC specimens, consistent with our previous study that showed that high SPHK1 expression was positively correlated with clinical stage and locoregional recurrence. These results provide new insights into the mechanism by which SPHK1 overexpression contributes to the development and progression of NPC and suggest that targeting SPHK1 might be a potential therapeutic strategy for NPC.

In conclusion, we found that inhibiting SPHK1 using specific siRNAs or the pharmacological inhibitor FTY720 led to potent anti-cancer activity in NPC *in vitro* and *in vivo*. Further preclinical and clinical studies aimed at developing SPHK inhibitors, particularly FTY720, are warranted to treat NPC.

## MATERIALS AND METHODS

### Cell lines and treatments

The human NPC cell lines CNE-1 and CNE-2 (ATCC, Manassas, VA, USA) were cultured in RPMI 1640 medium (Gibco, Grand Island, NY, USA) supplemented with 10% fetal bovine serum (Gibco). The immortalized normal human nasopharyngeal epithelial cell line, NP69, was cultured in defined-KSFM medium with epidermal growth factor (EGF) (Invitrogen, Camarillo, CA, USA). Cells were incubated in a humidified chamber with 5% CO_2_ at 37°C. During the experiment, CNE-1 and CNE-2 cells were treated with either diluted FTY720 (Sigma, St. Louis, MO, USA) or SPHK1 siRNA using Lipofectamine 2000 (Invitrogen) for transfection.

### Tissue specimens

A total of 142 archived paraffin-embedded NPC samples were used for the immunohistochemical analysis, as described in our previous report [19]. This study was approved and supervised by the ethical committee of the Southwest Hospital.

### RNA isolation and quantitative real-time PCR (qRT-PCR)

Total RNA was extracted from tumor cells using RNAiso (TaKaRa, Dalian, China). qRT-PCR was performed as previously described [21]. The primer sequences of are as follows: SPHK1 forward primer 5′-CTTGCAGCTCTTCCGGAGTC-3′, SPHK1 reverse primer 5′-GCTCAGTGAGCATCAGCGTG-3′, β-actin forward primer 5′-GACAGGATGCAGAA GGAGATTACT-3′, β-actin reverse primer 5′-TGATCC ACATCTGCTGGAAGGT-3′.

### Western blot analysis

Protein extracts were separated by electrophoresis in sodium dodecyl sulfate (SDS)-polyacrylamide gels (Invitrogen) and then transferred onto polyvinylidene fluoride membranes (Millipore Biotechnology, Billerica, MA, USA) for immunoblotting. The membranes were incubated first with a primary antibody at 4°C overnight and then with a horseradish peroxidase (HRP)-conjugated secondary antibody for 1 h at room temperature. The following antibodies were used: SPHK1 (Abcam, Cambridge, UK), Akt, p-Akt (Ser473), caspase-3 (Cell Signaling Technology, MA, USA), GAPDH, MMP-2, MMP-9, ERK, and p-ERK (Santa Cruz, CA, USA). Bands were visualized using an enhanced chemiluminescence (ECL) kit (Millipore) according to the manufacturer's protocol.

### Immunohistochemical staining

Immunohistochemistry was performed as previously described [21]. The following antibodies were used: SPHK1, Ki-67(Abcam), p-Akt (Ser473), and caspase-3 (Cell Signaling Technology). The morphological evaluation of necrosis grade was performed using hematoxylin/eosin (H&E)-stained slides, which were semiquantitatively scored (0: absence; 1: low level; 2: intermediate level; and 3: high level). Ki-67-positive cells were defined as those with brown staining in the nucleus, and the expression of Ki-67 was evaluated based on the percentage of positive tumor cells out of 1000 tumor cells. SPHK1-, p-Akt-, and caspase-3-positive cells were defined as those with immunoreactivity in both the cytoplasm and the nucleus. The expression of these markers was quantified using a composite score that was obtained by multiplying the scores for the staining intensities (0, no staining; 1, weak staining; 2, moderate staining; and 3, strong staining) by the scores for the percentage of positive cells (0, 0%; 1, < 10%; 2, 10–50%; 3, > 50%). To statistically analyze the data, the tumor sample cohort was grouped with the low-expression (≤ 4) and high-expression (≥ 6) cohorts.

### Transfection and vector construction

SPHK1-specific small interfering RNAs (si-SPHK1-1: 5′-GGGCAAGGCCUUGCAGCUCd(TT)-3′ and 5′-GAGCUGCAAGGCCUUGCCCd(TT)-3′; si-SPH K1-2: 5′-GCAGCUUCCUUGAACCAUUd(TT)-3′ and 5′-AAUGGUUCAAGGAAGCUGCd(TT)-3′) or control siRNA 5′-UUCUCCGAACGUGUCACGUd(TT)-3′ and 5′- ACGUGACACGUUCGGAGAAd(TT)-3′ were transiently transfected using Lipofectamine 2000 (Invitrogen) according to the manufacturer's instructions. The SPHK1-expressing lentivirus was constructed by inserting the SPHK1 open reading frame into the pLenti6.3 vector (Invitrogen), as previously described [21].

### Cell proliferation, viability, cell cycle and apoptosis assays

Cell proliferation and viability were measured using WST-8 staining with a Cell Counting Kit-8 (Dojinodo, Shanghai, China) according to the manufacturer's instructions. Cell cycle and apoptosis assays were performed using flow-cytometry analysis as previously described [21].

### Wound-healing assay and migration assay

Cells (1 × 10^6^/well) were seeded in six-well plates. Upon reaching the appropriate confluence, monolayers of cells were scratched using a sterile plastic tip. Images were captured at different time points (0, 24 h and 48 h) under a microscope to assess the rate of gap closure. Cell migration was measured using a transwell chamber (24-well insert, 8-μm pore size, Millipore). CNE-1 and CNE-2 cells were suspended in serum-free DMEM medium containing 0.1% BSA. Then, 0.2 ml of the cell suspension (5 × 10^4^ cells) was added to the top of the Transwell chambers, and the lower chamber was filled with 10% fetal bovine serum as a chemoattractant. The cells were then incubated for 24 h. FTY720 was added to both chambers and wells. At the end of the experiment, the non-invading cells on the upper surface of the membrane were removed, and the cells on the lower surface were fixed and stained using crystal violet. Five visual fields in each chamber were randomly chosen for cell counting under a light microscope.

### Sphingosine kinase assay

SPHK1 enzymatic activity was measured using a commercial Sphingosine Kinase Activity Assay Kit (Echelon, Salt Lake City, UT, USA) according to the manufacturer's instructions [18].

### Animal study

The animal experiments were performed as previously described [20]. Five-week-old female BALB/c-nu mice were purchased from the Peking University Animal Center (Beijing, China). After the mice were allowed to acclimatize for 5 days, they were subcutaneously injected in the right thigh with 1 × 10^6^ CNE-2 cells. Twenty days after implantation, the mice were randomly divided into different groups and treated for eighteen days with daily intraperitoneal injections (i.p.) of PBS (control), 2.5 mg/kg FTY720, 5 sessions of radiation (4 Gy) that were applied every 3 days, or a combination of the two treatments. Two days after the last treatment, all of the mice were sacrificed. Tumor volume was calculated using the equation (L × W^2^)/2, and tumor weights were measured and recorded in grams. The tumors were then collected and processed for H&E and immunohistochemical analysis or for SPHK1 activity analyses. All studies were approved by the Institutional Animal Care and Use Committee of Third Military Medical University.

### Statistical analysis

The statistical significance of differences between groups in the *in vitro* and *in vivo* assays were evaluated using the Mann-Whitney *U* test. All statistical tests were two-sided, and *p* values < 0.05 were considered significant. Calculations were performed using GraphPad Prism Software (www.graphpad.com).

## SUPPLEMENTARY MATERIALS


