# Supplementary material for: Sphingosine kinase 1 is a potential therapeutic target for nasopharyngeal carcinoma
Source: Oncotarget. 2016 Nov 2;7(49):80586–98. doi: 10.18632/oncotarget.13014 (PMC5348343; doi:10.18632/oncotarget.13014)
Supplement: Supplementary file 1 [file oncotarget-07-80586-s001.pdf]

Sphingosine kinase 1 is a potential therapeutic target for nasopharyngeal carcinoma

Supplementary Materials

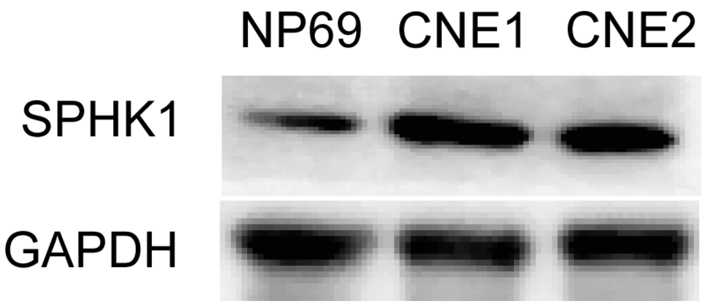

Supplementary Figure S1: Western blot analysis of the expression of SPHK1 in NP69 immortalized normal human nasopharyngeal epithelial cells and NPC cell lines; GAPDH was used as a loading control.
